# The development of nasal polyps involves early middle meatus mucous remodeling via TGF-β1 mediated PAI-1 reduction^☆^

**DOI:** 10.1016/j.bjorl.2023.01.007

**Published:** 2023-02-14

**Authors:** Yijun Liu, Longlan Shu, Xiaocong Jiang, Yue Zhang, Qian Chen, Yang Shen, Yucheng Yang

**Affiliations:** The First Affiliated Hospital of Chongqing Medical University, Department of Otorhinolaryngology, Chongqing, China

**Keywords:** Tissue remodeling, Fibrosis, Early nasal polyps, TGF-β1, Tissue culture in vitro

## Abstract

**Objective:**

Our study aimed to elucidate the effect of PAI-1 (Plasminogen Activator Inhibitor-1) and t-PA (Tissue-type Plasminogen Activator) in tissue remodeling in nasal polyps patients.

**Methods:**

Samples were streamed as early Nasal Polyps (eNP, n = 10) and inferior tissue from the same patient, mature Nasal Polyps (mNP, n = 14), and Control group (n = 15), respectively. Immunohistochemistry and immunofluorescence were applied to detect localization. Quantitative Real-Time Polymerase Chain Reaction (qRT-PCR) and Western blot were used to measure different levels among three groups. The mNP tissue was cultured in vitro and treated with TGF-β1 (Transforming Growth Factor-beta 1) activator, TGF-β1 inhibitor (SB431542), and PAI-1 inhibitor (TM5275); then Western blot, qRT-PCR, and ELISA were used to assess changes.

**Results:**

The immunohistochemistry and immunofluorescence showed that PAI-1 expression decreased in eNP and mNP, mainly in epithelium and glands. The transcriptional expression and protein level of TGF-β1/t-PA/PAI-1/Collagen1 were lower in eNP than IT while mNP group demonstrated lower mRNA expression and protein level of TGF-β1/t-PA/PAI-1/Collagen1 than Control group. In mNP tissue culture in vitro, TGF-β1 activator elevated t-PA, PAI-1, and Collagen1 with higher release of PAI-1 and Collagen1 in supernatant, whereas SB431542 suppressed above reactions; TM5275 lowered transcriptional and protein level of Collagen1 in supernatant.

**Conclusion:**

Early Nasal polyps’ formation in middle meatus mucous is related with fibrillation system PAI-1/t-PA and tissue remodeling; moreover, nasal polyps’ development is regulated by TGF-β1-mediated PAI-1 reduction.

**Level of evidence:**

3b.

## Highlights

• We have investigated the fibrosis genes in the TGF-β pathway in the early and mature nasal polyps development process, respectively.
• PAI-1 is involved in early nasal polyps, and its low fibrolysis is predisposed to nasal polyps’ development.
• PAI-1 is affected by the TGF pathway, and low PAI-1 expression is one of the main reasons for tissue remodeling in nasal polyps.

## Introduction

Chronic Rhinosinusitis (CRS) is a complicated sinonasal inflammation disease with a prevalence of 5%–12% in adults.^1^ CRS with Nasal Polyps (CRSwNP) is the most common phenotype in practice and visualize with mucosal edema and hyperplasia and localized fibrosis.^2^ Moreover, CRSwNP patients suffer from more severe symptoms and a high two-year recurrence rate of 55.3%, causing a decline in quality of life.^3^, ^4^ The underlying mechanism remains unclear to this day; however, it is generally acknowledged that tissue remodeling and mucous inflammation are vital in the formation and development of CRSwNP.^5^ Some research proved that those two factors construct a complex and dynamic network in early and mature nasal polyps development, and each stage has unique pathological features.^6^ Besides, CRSwNP demonstrated two prominent histological features ‒ localized mucous edema and inadequate fibrosis. Some studies have also shown that TGF-β1 (Transforming Growth Factor-beta 1) and Collagen1 in Extracellular Matrix (ECM) are relatively deficient in Mnp,^4^, ^7^ but it needs further research to decide whether the same scenario happens in eNP.

TGF-β1 is a pleiotropic cytokine, and its typical upstream is the Smad-dependent signaling pathway.^8^ TGF-β1 is widely involved with upper respiratory disease tissue remodeling and acts as the master switch in the development of CRS.^9^, ^10^, ^11^ In the pathological deformity of CRSwNP, TGF-β1 participates in the initiation and maintenance of tissue fibrosis by inhibiting the release of pro-inflammatory mediators to alleviate tissue damage, activating fibroblasts to close wounds or causing abnormal collagen deposition in different organs.^9^, ^12^ Notably, TGF-β1 deficiency and pSmad2-positive cells in CRSwNP are dramatically lower than in control groups, resulting in inadequate tissue repair and local connective tissue sparing and edema.^1^, ^7^, ^13^, ^14^

Fibrotic receptors and inhibitors play key roles in tissue remodeling and inflammation.^1^ Different phenotypes and endotypes are associated with tissue remodeling in upper respiratory diseases.^15^ PAI-1 (Plasminogen Activator Inhibitor-1) is a member of the serine protease inhibitor family, primarily grouped as t-PA (Tissue-type Plasminogen Activator) and u-PA (Urokinase-type Plasminogen Activator), which assists in transforming fibrinogen into fibrin to take part in fibrolysis^4^; t-PA is also regarded as a central fibrinogen activator of fibrinolysis.^16^ PAI-1 is expressed in multiple organs,^17^ like skin, esophagus, lung, kidney, liver, eye, and heart.PAI-1 participates in dynamic disease procedures and expresses at a high level in hepatic fibrosis, mouse tissue homogenate, and fibroblasts over time.^18^ However, whether and how it is involved in the occurrence of CRSwNP, particularly in eNP, needs in-depth exploration.

PAI-1 is located in the downstream of TGF-β1 and TGF-β1/Smad induced elevating PAI level and skin fibrosis^17^ and it is highly expressed in some reversible lower respiratory diseases, like asthma.^19^, ^20^ Among many genes modulated by TGF-β1,higher PAI-1 levels facilitate tissue fibrosis in asthma patients.^19^, ^20^, ^21^ TGF-β1 induced the expression of PAI-1 as part of the TGF-β1-activated esophageal fibrosis network.^21^ All previous studies demonstrated that TGF-β1 regulated genetic change of its downstream PAI-1, but its role in CRS is yet to know.

The aim of this study is to investigate whether PAI-1/t-PA is involved in mucosal tissue remodeling in the early stage of nasal polyps, and to further explore the expression and mechanism of TGF-β1/PAI-1/Col-1 axis in mNP, to provide insights for precise clinical treatment of nasal polyps.

## Patients and methods

### Subjects’ classification and selection

#### Subjects’ classification

We define early nasal polyps based on the size of nasal polyps under nasal endoscopy, namely Lund-Kennedy scale (L—K scale). Score 1 point in l-K scale is termed as early nasal polyps which means nasal polyps only occur in the middle meatus. Score more than 1 (>1) point l-K scale indicating that nasal polyps beyond the middle meatus are termed mature nasal polyps. Early nasal polyps (eNP, n = 10) and mature nasal polyps (mNP, n = 14) were obtained from those who had been confirmed in preoperative diagnosis and required endoscopic sinus surgery. Tissues of the Control group (Control, n = 15) were operative patients diagnosed with inferior turbinate hypertrophy without any sinus disease. All specimens were obtained under general anesthesia.

#### Subjects’ selection

Subjects were selected from 30th October 2020 to 31st December 2021, and patients with nasal polyps were referred to the 2020 European Position paper on sinusitis and nasal polyps (EPOS2020), all confirmed by pathological report before surgery. The enrollment criteria included: (1) Patients without any prior sinus surgery; (2) Patients who do not suffer lower respiratory diseases, such as asthma and chronic bronchitis; (3) Patients who do not take any relevant medications, such as NSAID or anti-coagulants. Endoscopy, sinus CT scan, and clinical symptom assessment were marked by Lund–Kennedy Scale, Lund–Mackay, and Visual Analog Scale (VAS) respectively. Detailed information are shown in Table 1. This research protocol was approved by the ethics committee of the First Affiliated Hospital of Chongqing Medical University and patient informed consent was obtained prior to the study.

**Table 1:** Clinical characteristics of the patients.

	Control	eNP	mNP	p
Number of patients	15	10	14	
Age, year	41 (34–60)	34 (28–49)	38 (32–57)	NS
Gender, male/female	8/7	5/5	6/8	NS
Positive skin prick test n/N	0/15	0/10	0/14	NS
Asthma n/N	0/15	0/10	0/14	NS
Endoscopy score	0	1 (1‒1)	3 (2–4)	<0.05
CT score	0	4 (0–10)	14 (10–18)	<0.05
VAS score	2 (0–4)	5 (2–8)	8 (4–10)	<0.05

Data presented as medians and interquartile ranges. p-value represents comparisons between the eNP and mNP groups, obtained by Student’s *t*-test.

### Tissue distribution and preparation

Tissues were rinsed with PBS three times after surgery and divided into three sections. One was immediately applied for tissue culture in vitro; the other part was embedded in paraffin and prepared for thickness of 4–5 μm for Immunofluorescence (IF) and Immunohistochemistry (IHC); the last part was stored at −80 °C for Qualitative Real-Time PCR (q RT-PCR) and Western Blot (WB).

## Tissue culture and treatment in vitro

Specimens were cut into 4‒5 mm^3^ sections to pass through the filter, rinsed with PBS three times. Small fractions were transferred to a T-25 flask contained with tissue medium (Gibco) mixed with 10% Fetal Bovine Serum (FBS, Gibco) and 1% penicillin-streptomycin (Gibco). Tissues were suspended and incubated at 37 °C and 5% CO_2_ for 24 h. The processed tissues were equally distributed into a 6-well plate and each group was treated with a TGF-β1 activator 10 ng/mL (MCE), a TGF-β1 inhibitor (SB431542) 50 nM (MCE), and a PAI-1 inhibitor (TM5275) 75 μM (TargetMol), respectively. After 72 h’ stimulation, tissues were collected for WB and q RT-PCR, and supernatants for ELISA assay.

### Immunohistochemistry

After deparaffinized by gliding scale ethanol, the specimens were blocked by goat serum albumin from the general SP kit (ZSGB-Bioscience) and then incubated with primary antibodies. After a whole night, the samples were incubated in the presence of Biotin-labeled Goat anti-Rabbit IgG polymer. Next, the specimens were incubated for 15 more mins with streptavidin-peroxidase and then incubated with DAB (ZSGB-Bioscience), showing yellowish or brown precipitate, and proving positive. Finally, slides were counterstained with Hematoxylin eosin, washed thoroughly under running tap water, and sealed for endoscopic examination.

### Immunofluorescence

The paraffin sections were deparaffinized, permeabilized with 0.5% Triton X-100 (Beyotime), blocked with 10.0% goat serum albumin (Beyotime) for 1 hour, and incubated with primary antibodies. Twelve to fourteen hours later, the sections were incubated with fluorescent antibody Goat Anti-Rabbit IgG (1:300; Orange red DyLight 594 fluorescent markers; Abbkine) in darkness for 1 h. Specimens were counterstained using DAPI (Beyotime), sealed with nasal polish, and visualized under a fluorescence microscope.

### Masson trichrome staining

The paraffin sections were stained with Masson trichrome and visualized under an endoscope with 5–8 representative fields (×400 magnification), and ImageJ software was used to calculate the fibrotic area and collagen volume fraction.

### Qualitative real-time PCR

RNA in tissue was extracted by TRIzol (Invitrogen); then, cDNA reverse transcription was performed, to finally get cDNA, stored at −20 °C. The primer sequences were designed and synthesized by Takara, and the primer names and sequences are shown in Table 2. PCR protocol contained one cycle at 95 °C for 5 min. and the following 40 cycles at 95 °C for 15 s and at 60 °C for 30 s.

**Table 2:** Primer sets for PCR amplification.

Gene	Sequence
Human TGF-β1	Forward: CAGCAACAATTCCTGGCGATAC
	Reverse: GCTAAGGCGAAAGCCCTCAAT
Human PAI-1	Forward: GTGCTGGTGAATGCCCTCTAC
	Reverse: CAGTGCTGCCGTCTGATTTG
Human t-PA	Forward: GCTACTTTGGGAATGGGTCA
	Reverse: TGCTGTGTAAACCTTGCCTATC
Human Smad2	Forward: GAAACCTTCCA TGCA TCACAGC
	Reverse: CTTCTTGTCA TTTCTACCGTGGC
Human Smad3	Forward: ACTAACTTCCCCGCAGGCA TC
	Reverse: TGCTGTGGTTCA TCTGGTGGTC
Human Col-1	Forward: AAGGTGTTGTGCGATGACG
	Reverse: GGTTTCTTGGTCGGTGGGT
Human GAPDH	Forward: TTCTCTTGTGACAAAGTGGA
	Reverse: TGCTGACAATCTTGAGGGAG

### Western blot

Homogenates from tissue were lysed with RIPA buffer and quantitated by BCA assay reagent (Beyotime). Proteins were separated by SDS-PAGE, transferred onto PVDF membranes, and then blocked with 7% skim milk for 70 min. The membranes were incubated with following specific primary antibodies on an orbital shaker overnight: anti-PAI-1 polyclonal (1:1500, Abcam), anti-t-PA monoclonal (1:3000, Abcam), anti-TGF-β1 polyclonal (1:1000, Cell Signaling Technology, CST), anti-pSmad2/3 polyclonal (1:1000, Bioss), and anti-Col-1 monoclonal (1:1000, CST). The following day, the membranes were incubated with anti-rabbit antibodies (1:4000; Zen-Bioscience), and then visualized by the ECL system with a mixture of liquid A and B (A:B = 1:1, Zen-Bioscience). Eventually the images were analyzed by the ImageJ software.

### ELISA

After 72 h of treatment with TGF-β1 activator, SB431542, and TM5275, the concentration of secreted PAI-1 and Collagen1 of approximately 500 μL supernatant collected from every group was determined using an ELISA kit following the protocol.

### Statistical analysis

All data were presented as the mean ± SEM, and the analyses were done using GraphPad Prism 8. All data were tested for normality and Chi-Square. If data obeyed a normal distribution with equal variances, one-way ANOVA was used for comparison between groups. If not, Kruskal–Wallis *H*-test was executed to compare the relationship among variables; *p* < 0.05 was considered statistically significant.

## Results

### Clinical characteristics of patients

As shown in Table 1, the nasal endoscopy and CT scan in the eNP group were lower than those in the mNP group (*p* < 0.05) while there was no significant difference in age, gender, skin prick test, and asthma between the two groups. Using the Visual Analogue Scale for further comparison, there was a significant difference shown in *t*-test. In contrast to self-reported problems of patients with early nasal polyps, patients with mature nasal polyps complained more severe symptoms in our study.

### The expression of TGF-β1/PAI-1/ t-PA/Collagen-1 in eNP

Hematoxylin-Eosin Staining (HE)and immunohistochemistry showed edema and inflammatory cell infiltration in eNP (Fig. 2A), and IT demonstrated higher epithelial layers and less inflammatory cell infiltration. Immunofluorescence (Fig. 2B‒C) indicated that PAI-1/t-PA were mainly expressed in epithelium and glands. qRT-PCR (Fig. 2D) and Western blot (Fig. 2E) suggested that the mRNA expression and protein levels of TGF-β1/PAI-1/t-PA/Collagen1 were lower in the eNP group than in IT.

### The expression of TGF-β1/PAI-1/t-PA/Collagen-1 in control group and mNP

Mucous from mNP group turned edematous and loose with inflammatory cell infiltration and had thinner epithelium in HE and immunohistochemistry (Fig. 3A). In contrast with the Control group, immunofluorescence proved that the mean fluorescence intensity of PAI-1/t-PA was lower in the mNP group (Fig. 3B‒C). Interestingly, data obtained from qRT-PCR (Fig. 3D) and Western blot (Fig. 3E) suggested that the expression levels of TGF-β1/PAI-1/t-PA/Collagen1 were attenuated in the mNP group than in the Control group.

### TGF-β1 induced PAI-1 gene expression in tissue culture in vitro

To further investigate whether t-PA and PAI-1 are modulated by their upstream TGF-β1signaling pathway, we conducted a tissue stimulation experiment in vitro. The qRT-PCR results showed that the expression levels of Smad3/ PAI-1/t-PA were higher in the mNP group than in the Control group after being stimulated with the TGF-β1 activator, whereas the SB431542 treated mNP group showed opposite results, and Smad2 mRNA expression did not change (Fig. 4A).The Western blot and ELISA findings showed that the mNP group treated with the TGF-β1 activator had higher expression levels of PAI-1/t-PA than the Control group; consistently, mNP tissue stimulated by SB431542 showed contrary results (Fig. 4B‒C).

### PAI-1 takes part in TGF-β1-mediated expression of fibrosis gene Collagen1

The results of qRT-PCR, Western blot, and ELISA indicated that the TGF-β1 activator elevated the mRNA expression, protein level, and supernatant secretion of Collagen1 in the mNP group. Moreover, Collagen1 expression suggested the opposite trend after being stimulated with SB431542 and TM5275 (Fig. 5A‒C).

## Discussion

**Figure 1:**
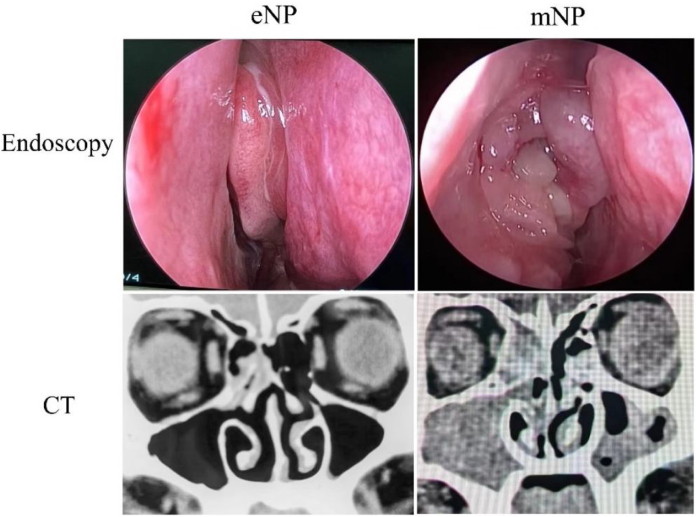
Representative images of eNP and mNP in endoscope and CT scan.

CRS is a heterogeneous disease involving multiple cells and inflammatory cytokines with changeable pathophysiological features due to different endotypes and phenotypes.^22^ Studies have shown that the diagnosis of early nasal polyps is of great clinical significance for treatment and prognosis. As shown in Fig. 1 and Table 1, the invasion of eNP was found to be much smaller than that mNP by nasal endoscopy and CT scan, and the scores of nasal endoscopy and CT also showed that eNP was lower than mNP.

**Figure 2:**
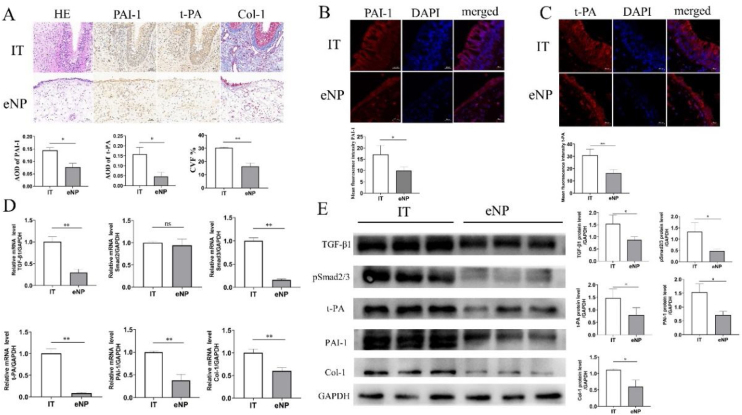
Localization and quantitative expression of t-PA and PAI-1 in eNP and IT. (A) Representative images hematoxylin eosin stain (HE), Immunohistochemistry, and Masson Trichrome stain. AOD, average optical density; CVF, collagen volume fraction. (B, C) Immunofluorescence images of t-PA and PAI-1 in eNP and IT (×400 magnification). (D) qRT -PCR and (E) Western blot showed that the mRNA expression and protein levels of t-PA, PAI-1, and Col-1 were higher in IT than in eNP. *p < 0.05, **p < 0.01, ***p < 0.001 (300 × 300 DPI).

**Figure 3:**
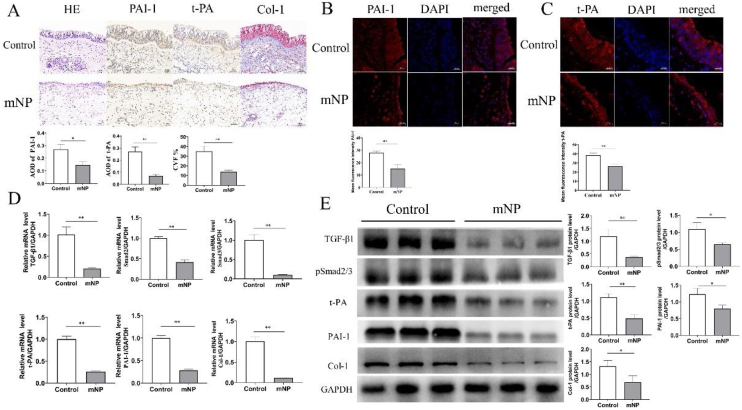
The position and quantification expression of PAI-1 and t-PA in the Control and mNP group respectively. (A) Representative Immunohistochemistry images of PAI-1 and t-PA in the Control and mNP groups. (B, C) Immunofluorescence representative images (×400 magnification). (D) qRT-PCR and (E) Western blot indicated that the mRNA expression and protein levels of t-PA, PAI-1, and Col- 1. *p < 0.05, **p < 0.01, ***p < 0.001 (300 × 300 DPI).

**Figure 4:**
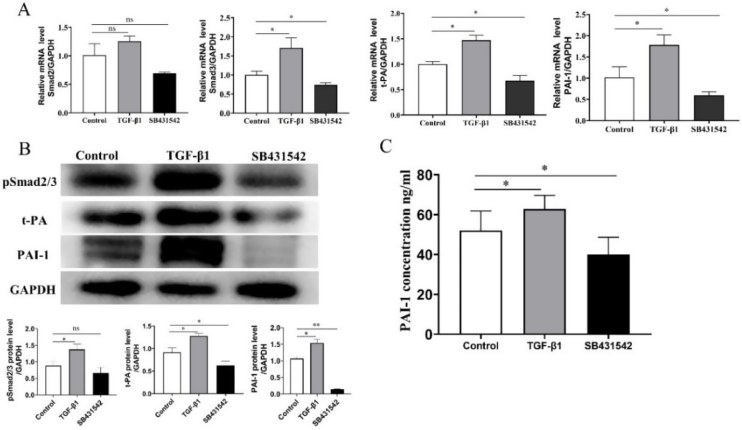
Mature nasal polyps tissue culture treated with TGF-β1 activator elevated the (A) mRNA expression and (B) protein levels of t-PA, PAI-1, pSmad2/3, and (C)increased the release of PAI-1 in the supernatant. Consistently, the opposite trend was observed after SB431542 treatment in vitro tissue culture. (300 × 300 DPI).

**Figure 5:**
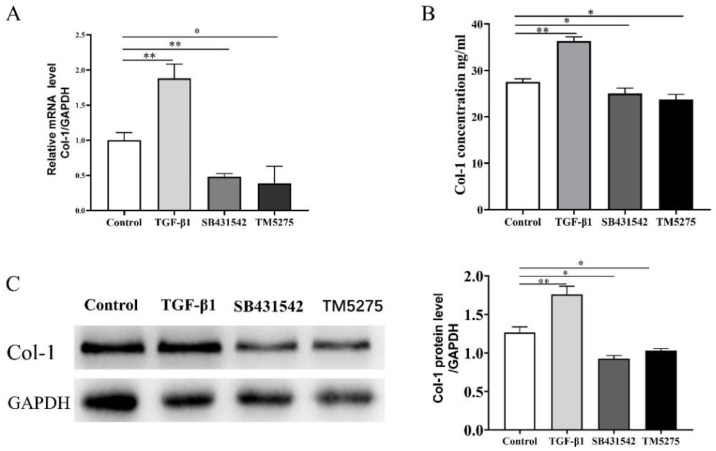
Mature nasal polyps tissue culture in vitro treated with TM5275, the results presented the reduction of (A)Col-1 mRNA expression, (B) Col-1 secretion in supernatant, and (C) protein level. (300 × 300 DPI).

Our findings confirmed that TGF-β1 induced PAI-1 reduction in CRS. Compared with IT, the mRNA expression and protein level of TGF-β1/PAI-1/t-PA/Collagen1 was lower in eNP group, while Smad2 presented no statistical significance, which is in line with predilection site of nasal polyps in uncinate tissue in the middle meatus. PAI-1 is a 43 kDa (≈50 kDa) glycosylated secreted protein that effectively regulates the fibrinolytic system by inhibiting serine protease, t-PA, and u-PA, which is a key mediator in disease occurrence and development. Compared to mature ethmoidal polyps and normal nasal mucous, eNP showed a more severe epithelial loss, including epithelial damage, repair responses, and tissue remodeling, which resembled the tissue remodeling that occurs in parallel with local inflammation instead of happening later.^6^ While our research primarily focused on the dynamic process of tissue remodeling in CRS. It also emphasized that PAI-1 is a vital mediator throughout the occurrence and development of nasal polyps. Consequently, we speculated that low expression of TGF-β1/ PAI-1/t-PA/Collagen1 in eNP contributed to insufficient repair after epithelial injury, thereby developing into mature nasal polyps.

Beyond that, we made great efforts to explore the development process of mNP. TGF-β1, an important fibrogenic factor,^13^ regulates the transcriptional and expression levels of various downstream genes via the Smad signaling pathway. A previous study reported that elevated TGF-β1 expression and reduced Smad7 expression were found in the fibrotic myocardial tissue of hypertensive rats. In previous studies, low levels of TGF-β1 in CRSwNP contributed to tissue repair and collagen reduction, which is one of the leading causes of the development of CRSwNP.^14^ Additionally, overexpression of PAI-1 promotes collagen accumulation while PAI-1 knockdown had lower collagen levels in skin fibroblasts, which strongly suggested that collagen was regulated by PAI-1. Higher concentrations of PAI-1 in endometriosis patients may inhibit u-PA activity, leading to collagen accumulation;^23^ indicating that abnormal PAI-1 tissue level is correlated with multiple diseases. Our study showed that lower PAI-1 alleviated local fibrosis in CRS and also confirmed the differential expression of PAI-1 in CRSwNP.

We assumed that collagen was regulated by PAI-1 and involved in tissue remodeling in CRS with PAI-1 located in the downstream of TGF-β1. In tissue culture in vitro treated with the TGF-β1 activator, the mRNA expressions and protein levels of PAI-1 and t-PA, and Collagen1 were higher than in the Control group, but SB431542 counterbalanced the results. Furthermore, TM5275 reduced both the transcriptional and protein levels of Collagen1, confirming that the expression level of PAI-1 correlated with fibrosis gene expression in mNP tissue remodeling and could be initiated by TGF-β1 as part of the tissue remodeling fibrosis network.^24^ Studies proved that oral administration of TM5275 to mice resulted in reduced collagen deposition in the airway.^21^, ^25^ Moreover, the plasma PAI-1 of patients diagnosed with eosinophilic esophagitis was positively linked with peripheral TGF-β1 levels.^21^ By stimulating mNP tissue culture in vitro, we confirmed that TGF-β1 regulated the changes of its downstream, PAI-1, and that PAI-1 regulated the production of TGFβ1-induced collagen. However, recent studies revealed the dual characteristics of PAI-1; PAI-1 deficiency and the overexpression of its downstream target u-PA promoted cardio selective fibrosis.^26^ As a result, further investigations are needed to investigate whether the TGF-β1 signaling pathway regulated PAI-1 reduction in CRS and explore the exact mechanism.

This study had some limitations. First, much time was spent on tissue-level treatment in vitro without detailing signaling pathway investigation. Second, TGF-β1 is typically time-dose dependent and can be expressed in different organs, but we used the regular routine of TGF-β1 without treating tissues on time and concentration gliding scale.

## Conclusion

The expression levels of PAI-1/t-PA/TGF-β1, and Collagen1 were lower in the dynamic process of eNP; in the mNP tissue treatment in vitro, TGF-β1 regulated the expression of PAI-1/t-PA, further modulating the fibrosis gene Collagen1 participation in tissue fibrosis and remodeling. PAI-1 could be a new potential marker of TGF-β1-induced PAI-1 reduction for mNP.

## Funding

10.13039/501100001809National Natural Science Foundation of China (grant no. 81970864), the Chongqing Middle and Youth Medical High-end Talent Studio Project (grant no. Yu Wei, 2018, no. 2), and the Chongqing Talent Project (grant no. cstc2021ycjh-bgzxm0080).

## Conflicts of interest

The authors declare no conflicts of interest.
